# Performance of non-laboratory staff for diagnostic testing and specimen collection in HIV programs: A systematic review and meta-analysis

**DOI:** 10.1371/journal.pone.0216277

**Published:** 2019-05-02

**Authors:** Lara Vojnov, Miriam Taegtmeyer, Caroline Boeke, Jessica Markby, Lindsay Harris, Meg Doherty, Trevor Peter, Nathan Ford

**Affiliations:** 1 Clinton Health Access Initiative, Boston, Massachusetts, United States of America; 2 Liverpool School of Tropical Medicine, Pembroke Place, Liverpool, United Kingdom; 3 World Health Organization, Geneva, Switzerland; National Institute for Communicable Disease Control and Prevention, SOUTH AFRICA

## Abstract

**Background:**

In most high HIV burden countries, many HIV patients do not have reliable access to required diagnostic laboratory tests. Task shifting of clinical tasks to lower cadres of health care workers and lay counselors has been successful in scaling up treatment for HIV and may also be an effective strategy in expanding access to essential diagnostic testing.

**Methods:**

We screened major electronic databases between 1 January 2005 to 26 August 2018 to identify studies assessing ease of use and accuracy of task shifting of HIV-related diagnostic testing and/or specimen collection to non-laboratory health staff. Two independent reviewers screened all titles and abstracts for studies that analyzed diagnostic accuracy, patient impact, ease-of-use, or cost-effectiveness. Studies were assessed for quality, bias, and applicability following the QUADAS-2 framework. We generated summary estimates using random-effects meta-analyses.

**Results:**

We identified 42 relevant studies. Overall, point-of-care CD4 testing performed by non-laboratory staff had a mean bias of -54.44 (95% CI: -72.40 –-36.48) compared to conventional laboratory-based. Though studies were limited, the diagnostic accuracy of point-of-care alanine transaminase enzyme (ALT) and hemoglobin testing performed by non-laboratory staff was comparable to conventional laboratory-based testing by laboratory professionals. Point-of-care testing and/or specimen collection were generally found to be acceptable and easy to use for non-laboratory staff.

**Conclusions:**

Task shifting of testing using point-of-care technologies to non-laboratory staff was comparable to laboratory professionals operating the same technology in the laboratory. Some variability was observed comparing the performance of point-of-care CD4 testing by non-laboratory staff to conventional laboratory-based technologies by laboratory professionals indicating potential lower performance was likely technological rather than operator caused. The benefits of task shifting of testing may outweigh any possible harms as task shifting allows for increased decentralization, access of specific diagnostics, and faster result delivery.

## Background

In most high HIV burden countries in sub-Saharan Africa, many HIV patients currently do not have reliable access to required diagnostic laboratory tests. This is in part because these countries have limited health human resource capacity, including laboratory professionals. The successful scale up and management of antiretroviral therapy in Africa and other low- and middle-income settings has depended critically on a public health approach that relies on decentralization of treatment to primary health care facilities and lesser-trained health workers in primary care services to deliver HIV testing and antiretroviral therapy to the majority of HIV positive patients [1]. This approach has supported the delivery of HIV testing and antiretroviral therapy at scale in resource-limited settings: of the estimated 19.4 million people with HIV receiving antiretroviral therapy, nearly 14 million are living in Africa [2,3]. Providing greater access to antiretroviral therapy and diagnostic testing are critical to achieve UNAIDS 90-90-90 targets by 2020 [4].

Task shifting describes the process of capacitating lesser-trained health workers to provide specific services previously delivered by specialists with higher levels of training. The severe shortage of health care workers including doctors, nurses, and laboratory professionals in high HIV burden countries led a number of countries in sub-Saharan Africa to pioneer task shifting and allow lay providers to conduct HIV rapid testing and nurses to deliver antiretroviral therapy, significantly expanding access to services [5–8]. Lay provider HIV testing was recommended in the 2015 WHO consolidated guidelines on HIV testing services following a systematic review of the evidence demonstrating non-inferiority [6,7,9]. Furthermore, nurse-led antiretroviral therapy delivery was endorsed by the World Health Organization in 2007 [8], with safety and efficacy subsequently validated through randomized trials since 2010 [10].

With increasing numbers of patients tested and on antiretroviral therapy, greater access to laboratory monitoring will be required to optimize treatment, prevent adverse outcomes, and reduce onward transmission. The lack of skilled laboratory professionals at health care facilities, particularly in rural settings, may similarly justify task shifting of diagnostic testing and specimen collection to lower cadres of health care workers. Despite rapid scale up of antiretroviral therapy services many countries face difficulties in diagnosing opportunistic infections and monitoring treatment safety and effectiveness due to a shortage of laboratory professionals and other skilled health workers to collect the specimens and conduct testing [11]. While WHO guidelines for the management of HIV infection have promoted an approach that relies on limited laboratory tests, there remains a need to ensure that a minimum set of tests are available to diagnose severe opportunistic infections and to monitor treatment safety and effectiveness [12]. A number of existing diagnostic technologies have been developed that can be used outside of sophisticated and specialized laboratory systems and allow for greater decentralization and wider access to diagnostic testing [13]. Rapid diagnostic tests for HIV and syphilis diagnosis and liver function testing as well as point-of-care technologies for CD4, HIV nucleic acid (viral load and early infant diagnosis), creatinine, and hemoglobin testing exist, with more in development [13]. In several countries, lower cadre health workers have been successfully trained to collect sputum and/or whole blood specimens and to conduct a range of discrete laboratory tests, including HIV/syphilis rapid tests as well as CD4 cell count measurements, TB, malaria, chemistry, liver function and hematology assays [14–55].

We undertook this systematic review in order to summarize the available evidence on task shifting of specimen collection and performing common laboratory tests using point-of-care technologies as part of monitoring routine HIV care and treatment.

## Methods

### Search strategy and study selection

This review was carried out according to the Preferred Reporting Items for Systematic Reviews and Meta-Analyses (PRISMA) [56]. The protocol was reviewed and approved by WHO. PubMed, Medline and EMBASE databases were searched from 1 January 2005 to 26 August 2018 in parallel to identify peer-reviewed original research. An initial search was performed on April 23^rd^, 2015 and an updated search on August 27^th^, 2018 –search terms were the same and the results combined. Search terms were developed using the MeSH term formats as follows: HIV-positive patients (population), non-laboratory professionals (operators), task shifting (intervention), and outcomes (accuracy, retention, cost-effectiveness, and acceptability) (S1 Fig). Conference abstracts within the search dates from the Conference on Retroviruses and Opportunistic Infections (CROI), International Conference on AIDS and STIs in Africa (ICASA), International AIDS Society (IAS), and AIDS Conference and bibliographies were also screened and reviewed for possible inclusion. Two reviewers (LV, CB, JM, LH) working independently screened all titles and abstracts for eligibility. Studies were included if they compared the diagnostic accuracy, patient impact, acceptability, or cost-effectiveness of non-laboratory staff performing HIV-related diagnostic testing and/or specimen collection compared to laboratory professionals. Bibliographies of all included studies were also reviewed to identify unpublished, non-peer reviewed work for possible inclusion. Non-English studies were excluded. Data were extracted from each included study including sample size, sample type, test setting, end-user cadre, comparator, study dates, and outcomes of diagnostic accuracy metrics and acceptability. Studies were assessed for quality, bias and applicability following the QUADAS-2 quality appraisal tool [57].

### Data analysis

The primary outcome of interest was the diagnostic accuracy of non-laboratory staff in performing the specimen collection and/or diagnostic test compared to laboratory professionals. In particular, bias and sensitivity/specificity were sought. Secondary outcomes included timing and retention of patients along the cascade of care, cost-effectiveness, and acceptability of testing or specimen collection when performed by non-laboratory staff compared to laboratory professionals. To determine the presence of between-study heterogeneity, the I-squared statistic was calculated [58]. When at least four studies exist, random effects models were used to estimate the pooled summary measures for diagnostic accuracy: the metaprop (for proportions) and metan (for continuous values) were used in Stata with a continuity correction value of 0.5 and exact confidence intervals.

Two reviewers (LV, CB/JM) independently performed the statistical analysis to ensure accuracy. Graphic representations were completed in GraphPad Prism v6.0 (La Jolla, California, USA) and analyses were completed in Stata 13 (College Station, Texas, USA).

## Results

### Study characteristics

**Fig 1:**
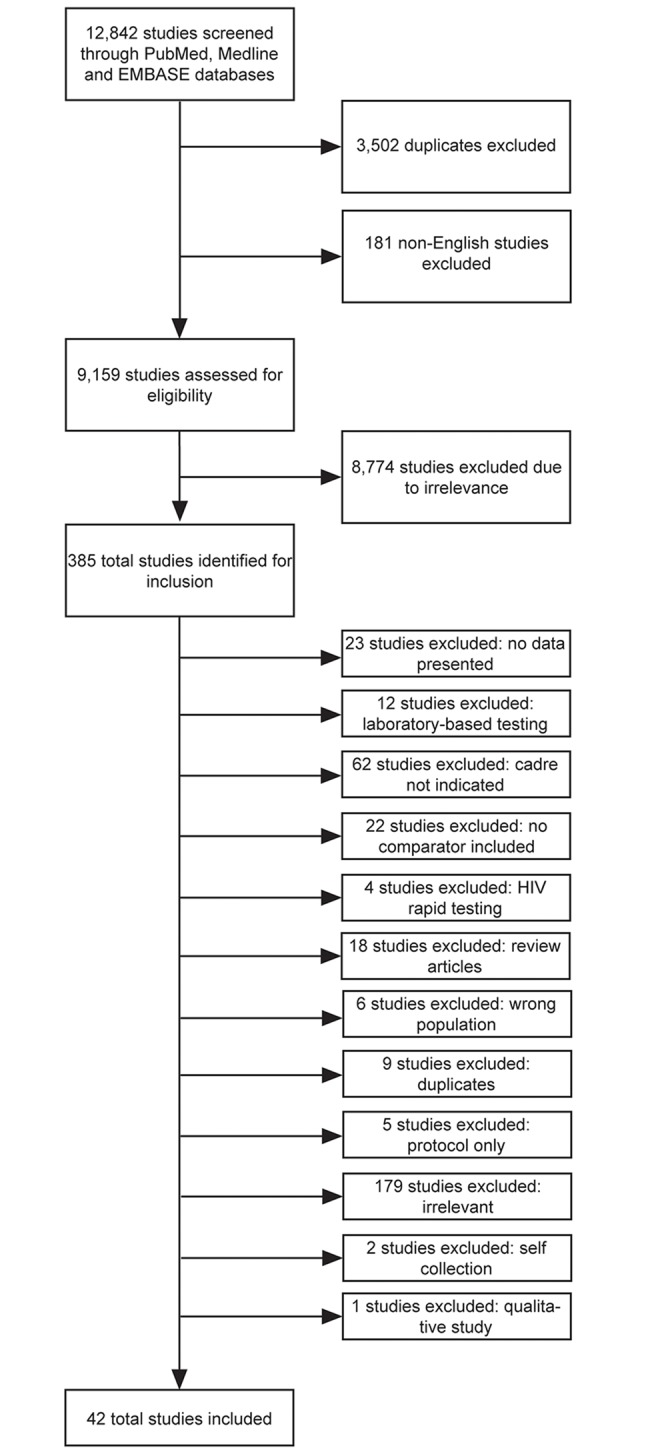
PRISMA flow chart.

Of 12,842 titles screened, 42 eligible studies were included for review (Fig 1) [14–55]. Approximately 13,686 data points were included in the diagnostic accuracy analysis and 43 different analyses. The included studies spanned 20 countries (Table 1). Most studies (86%) were carried out in Africa, three were performed in Brazil, and one each in Vietnam and China. Testing and specimen collection were performed at a mix of health care facilities: urban hospitals, urban clinics, urban outreach, remote hospitals, and rural clinics. Ten different point-of-care test types with 14 different technology assays were included performed by 11 non-laboratory health care cadres (Table 2); five studies analyzed specimen collection. Sixty-four percent of studies looked at POC CD4 with 85% of those studies reviewing the accuracy of the Alere Pima technology. Nurses were included as index test end-users in 60% of studies. All studies were observational or diagnostic accuracy studies, except for one randomized controlled trial and one meta-analysis. Studies were conducted between 2006 and 2018.

**Table 1:** Study characteristics of included studies in meta-analysis.

Author	Ref #	Journal	Year	Countries of study	Type of study	Years of study	Site type	Test	Technology	Sample type	Comparator
Agizew	[14]	PLoS One	2017	Botswana	step-wedged clinical trial	2012–2014	HIV C&T sites	POC TB/RIF	Xpert MTB/RIF	Sputum	Xpert MTB/RIF
Arnett	[15]	IAS poster	2013	Tanzania	technical evaluation	unknown	clinics	POC CD4	Alere Pima	unknown	Pima in lab
										finger-prick capillary	laboratory CD4
										finger-prick capillary microtube	laboratory CD4
										venous	laboratory CD4
Benzaken	[16]	STI	2014	Brazil	prospective observational	2010–2011	remote clinics	syphilis	TR Syphilis 3.0-SD Bioline	DTS (6 each)	known results
Bile	[17]	CROI poster	2017	Bostwana	randomised controlled trial	2013–2016	mobile and home testing	POC CD4	Alere Pima	unknown	laboratory Alere Pima
Brouillette	[18]	IAS poster	2013	Uganda	retrospective observational	2011–2012	clinics	POC CD4	Alere Pima		
Bwana	[19]	PLoS One	2015	Kenya	prospective observational	2014–2015	rural clinics, sub-district hospital	POC CD4	BD FACS Presto	finger-prick capillary	BD FACS Presto
											BD FACS Count
											BD FACS Calibur
											Alere Pima
Daneau	[20]	PLoS One	2016	Tanzania	prospective observational	2014	urban clinics	POC CD4	BD FACS Presto	finger-prick capillary	BD FACSCalibur
										venous blood	
Diaw	[21]	JAIDS	2011	Senegal	technical evaluation	2009–2010	urban clinics	POC CD4	Alere Pima	finger-prick capillary	BD FACSCount
Fajardo	[22]	Bulletin WHO	2015	9 sSA	retrospective observational	2011–2013	primary and mobile clinics, community	POC CD4	Alere Pima		NA
Garone	[23]	AIDS conf	2014	Malawi	prospective observational	2013–2014	primary clinic	viral load	dried blood spot	finger-prick capillary	NA
Gimbel-Sherr	[24]	HRH	2007	Mozambique	retrospective operational	2004–2005	urban clinics	POC CD4	not indicated	unknown	
Glencross	[25]	JIAS	2012	South Africa	technical evaluation	unknown	urban hospital	POC CD4	Alere Pima	finger-prick capillary	Beckman Coulter PLG
							urban clinic				
Gous	[26]	PLoS One	2013	South Africa	technical evaluation	2012	urban hospital	POC CD4	Alere Pima	finger-prick capillary	Beckman Coulter PLG
								Hemoglobin	HemoCue Hb201+		Advia 120, 2120
								ALT	Roche Reflotron Plus		Synchron DXC 800
								Cr	Roche Reflotron Plus		Synchron DXC 800
Gous	[27]	JAIDS	2016	South Africa	prospective observational	2010–2012	urban clinics	POC CD4	Alere Pima	venous blood / venidrop	Beckman Coulter
								Hemoglobin	HemoCue Hb201+		Advia 120 and 2120
								ALT	Roche Reflotron Plus		Advia 1800 and Synchron DXC 801
								Creatinine	Roche Reflotron Plus		Advia 1800 and Synchron DXC 804
								Lactate	Accutrend		Advia 1800
Jani	[28]	AIDS	2011	Mozambique	technical evaluation	2009–2010	urban clinics	POC CD4	Alere Pima	finger-prick capillary	BD FACSCalibur
								POC CD4	Alere Pima		Alere Pima in lab
								ALT	Roche Reflotron Plus		Selectra Junior
								AST	Roche Reflotron Plus		Selectra Junior
								Hemoglobin	HemoCue Hb201+		Sysmex SF3000
Jani	[29]	AIDS	2016	Mozambique	retrospective observational	2012–2013	clinics	POC CD4	Alere Pima	finger-prick capillary	NA
Kaindjee-Tjituka	[30]	Afr J Lab Med	2017	Namibia	prospective observational	2011	public clinics	POC CD4	Alere Pima	finger-prick capillary	NA
Kohatsu	[31]	PLoS One	2018	Tanzania	prospective observational	2011	urban clinics	POC CD4	Alere Pima	finger-prick capillary direct drop	BD FACSCalibur
										finger-prick capillary microtube	
										venous blood	
Lassovski	[32]	poster	2013	Swaziland	retrospective observational	2010–2012	rural/urban clinics	POC CD4	Alere Pima	controls	NA
								creatinine	Roche Reflotron Plus		
								glucose	Roche Reflotron Plus		
								potassium	Roche Reflotron Plus		
								ALAT	Roche Reflotron Plus		
Liang	[33]	Chin Med J	2015	China	prospective observational	2012	urban clinics	POC CD4	Alere Pima	finger-prick capillary	BD FACSCalibur
										venous blood	
MacLennan	[34]	AIDS	2007	Malawi	technical evaluation	2006	urban clinic	POC CD4	BD FACSCalibur	finger-prick capillary	venous FACSCalibur
Maiers	[35]		2014	South Africa	prospective observational		urban clinic		blood collection of 150ul	finger-prick capillary	
Manabe	[36]	PLoS One	2012	Uganda	technical evaluation	2009	urban hospital	POC CD4	Alere Pima	finger-prick capillary	
Morawski	[37]	JAIDS	2013	Uganda	technical evaluation		urban clinics	POC CD4	Alere Pima	venous blood	BD FACSCalibur
Mwanja	[40]	IAS poster	2013	Tanzania	retrospective observational	2011–2012	clinics	POC CD4	Alere Pima	finger-prick capillary	
Mwau	[41]	PLoS One	2014	Kenya	technical evaluation	2014	rural clinics	POC CD4	Zyomyx MyT4	finger-prick capillary	BD FACSCount
											Zyomyx in lab
Myer	[42]	JIAS	2013	South Africa	technical evaluation	unknown	urban clinic	POC CD4	Alere Pima	venous blood	laboratory assay
Negedu-Momoh	[43]	PLoS One	2017	Nigeria	prospective observational	2015–2016	rural hospital clinic	POC CD4	BD FACSPresto	finger-prick capillary	BD FACSCalibur
Olugbenga	[44]	PLoS One	2018	Nigeria	prospective observational		antenatal clinics	HIV/Syphilis	SD BIOLINE HIV/Syphilis	finger-prick capillary	SD BIOLINE HIV/Syphilis in lab
											TPHA Lab
Pinto	[45]	PLoS One	2015	Brazil	prospective observational	2013–2014	rural clinics	POC CD4	Alere Pima	finger-prick capillary	BD FACSCalibur
										venous blood	
Pollock	[46]	PLoS One	2013	Vietnam			urban clinic	ALT	Diagnostics for All	finger-prick capillary	Roche Cobas ALT
Riberio	[47]	STI	2014	Brazil	prospective observational	2011–2012	urban outreach	syphilis	SD Bioline Syphilis 3.0	DTS (4 each)	known results
Rutstein	[48]	JCV	2014	Malawi	technical evaluation	unknown	remote hospitals	viral load	dried blood spot	finger-prick capillary	NA
Sangala	[49]	IJTLD	2006	Malawi	qualitative	unknown	rural clinics, district hospital	tuberculosis	sputum collection	sputum	NA
Scott	[50]	BMC	2015	global	pooled data meta-analysis	2009–2014	various	CD4	Alere Pima	finger-prick capillary	conventional CD4
Simmonds	[51]	IAS poster	2018	Zimbabwe	retrospective observational	2016–2017		POC EID	Alere q HIV 1/2 Detect	heel-prick capllary	NA
Tsibolane	[62]	unpublished	2014	South Africa	retrospective observational	2014	urban/rural clinics	POC CD4	Alere Pima	finger-prick capillary	NA
Wake	[63]	JAIDS	2018	South Africa	prospective observational	2016–2018	HIV clinics	POC CrAg	CrAg LFA	finger-prick capillary	POC CrAG
Williams	[54]	Clin Infect Dis	2015	Uganda	prospective observational	2013–2014		POC CrAg	CrAg LFA	finger-prick capillary	POC CrAG
Zeh	[55]	J Immunol Methods	2017	Kenya	prospective observational	unknown	rural hospital clinic	POC CD4	Alere Pima	finger-prick capillary	BD FACSCalibur
										venous blood	BD FACSCalibur
										finger-prick capillary	Alere Pima in lab
Zinyowera	[38]	unpublished		Zimbabwe	prospective observational	unknown	central	POC viral load	SAMBA II	proficiency panel	known results
Zinyowera	[39]	JAIDS	2010	Zimbabwe	technical evaluation	unknown	urban clinic	POC CD4	Alere Pima	finger-prick capillary	BD FACSCalibur

C&T: care and treatment

ALT: alanine aminotransferase

AST: aspartate aminotransferase

DTS: dried tube specimens

Known results: proficiency panels with already known results

**Table 2:** Test types, technologies used, and non-laboratory health care cadres in included studies.

Test types and technologies used	Non-laboratory health care cadres
CD4: Alere Pima (device), Zyomyx MyT4 (device), BD FACSPresto (device)	Nurses (25 studies): staff, technicians, assistants, practitioners
Syphilis: SD Bioline (lateral flow)	Physicians (2)
HIV nucleic acid: dried blood spot (specimen), SAMBA II (device), Alere q HIV 1/2 Detect (device)	Health surveillance assistants (2)
ALT: Roche Reflotron Plus (device), Diagnostics for All (lateral flow)	ANC provider (2)
AST: Roche Reflotron Plus (device)	Clinic staff (7)
Hemoglobin: HemoCue Hb201+ (device)	Laypersons (1)
Creatinine, glucose, potassium: Roche Reflotron Plus (device)	Lay counsellors (6)
Tuberculosis: sputum collection (specimen), Cepheid GeneXpert MTB/Rif (device)	Biologists (2)
Cryptococcal antigen: IMMY CrAg LFA (lateral flow)	Microscopists (2)
Lactate: Accutrend (device)	VCT staff (1)
	Phlebotomist (1)

Overall, there was moderate risk of bias across the studies (S2 Fig). Patient, health care facility, and health care cadre inclusion and exclusion criteria were unclear or not stated in several studies. Studies often had variable study objectives and test types making comparisons across studies difficult. Only four diagnostic accuracy studies directly compared the technical performance of the point-of-care technology between non-laboratory staff and laboratory professionals. Twenty-six studies compared the accuracy of point-of-care CD4 testing performed by non-laboratory staff to laboratory CD4 assays performed by laboratory staff.

### Diagnostic accuracy of point-of-care testing performed by non-laboratory staff

**Fig 2:**
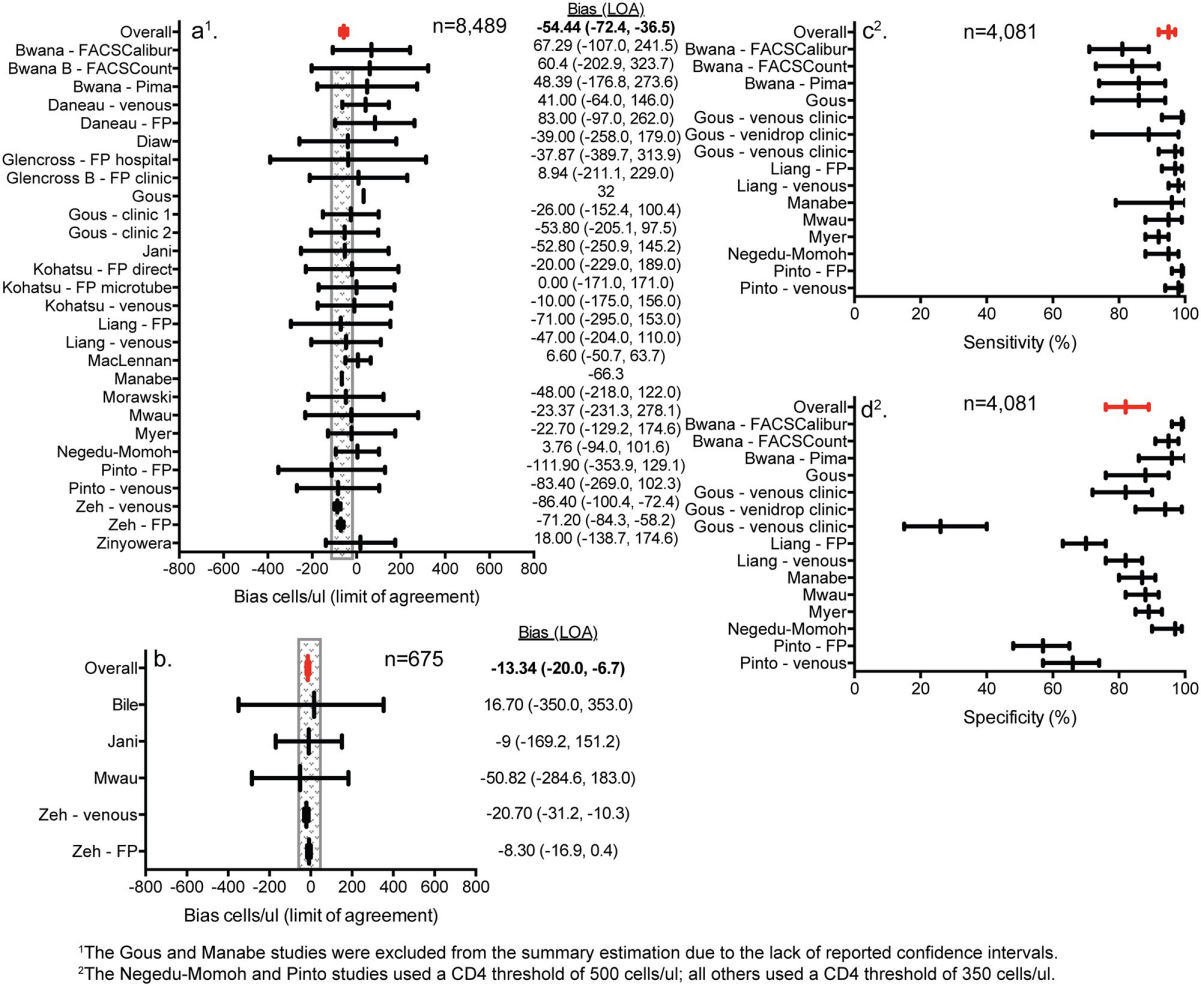
Forest plots of point-of-care CD4 diagnostic accuracy performance by non-laboratory staff. (a) point-of-care CD4 testing by non-laboratory staff compared to conventional laboratory-based testing by professional laboratory staff; (b) point-of-care CD4 testing by non-laboratory staff compared to point-of-care CD4 testing by professional laboratory staff; the shaded box represents +/- 50 cells/ul in (a) and (b). (c) sensitivity of correctly classifying patients below a CD4 threshold of 350 cells/ul; (d) specificity of correctly classifying patients above a CD4 threshold of 350 cells/ul.

The primary outcome observed across the majority of studies focused on the diagnostic accuracy performance of point-of-care testing when performed by non-laboratory staff. The mean bias measurement was most often included across studies [19–21,25,26,28,31,32,34,36,37,39,41–43,45,55]. Compared to conventional laboratory-based testing performed by laboratory professionals, point-of-care CD4 testing performed by non-laboratory staff had a mean bias of -54.44 (95% CI: -72.40 –-36.48) (I^2^: 17.6%, p = 0.212) (Fig 2a). Sixty-five percent (17/26) of studies had a mean bias within a +/- 50 cells/ul range. Only four studies compared the performance of point-of-care CD4 testing between laboratory professionals and non-laboratory staff [17,28,41,55]. The performance of each study was similarly within the +/-50 cells/ul range and the overall mean bias was -13.34 (95% CI: -19.98 –-6.69) (I^2^: 0.0%, p = 0.502) (Fig 2b). In one study, conventional laboratory-based testing performed by laboratory professionals had a coefficient of variation of approximately 7.5%, while the point-of-care CD4 technology performed by non-laboratory staff, nurses, had a coefficient of variation of approximately 10.7% [28]. The sensitivity and specificity of identifying patients in need of treatment based on the relevant CD4 count used at the time of the study was also calculated from relevant studies (Fig 2c and 2d). The estimated sensitivity and specificity were 95% (95% CI: 92–97%) (I^2^: 68.9%, p = 0.000) and 82% (95% CI: 76–89%) (I^2^: 95.6%, p = 0.000), respectively.

Two studies reviewed the performance of cryptococcal antigen lateral flow assays when used by non-laboratory staff [53,54]. The sensitivity and specificity of non-laboratory staff correctly identifying cryptococcal antigen were 100% in both studies. Additionally, syphilis testing by non-laboratory staff using the dual HIV/syphilis rapid diagnostic test had an agreement of 0.666 (0.358–0.974) and a specificity of 99.9% (95% CI: 99.8–100%) compared to when compared to laboratory technicians [44]. Furthermore, nursing staff successfully tested external quality assurance panels using syphilis rapid tests with a sensitivity and specificity over 90% [16,47].

**Fig 3:**
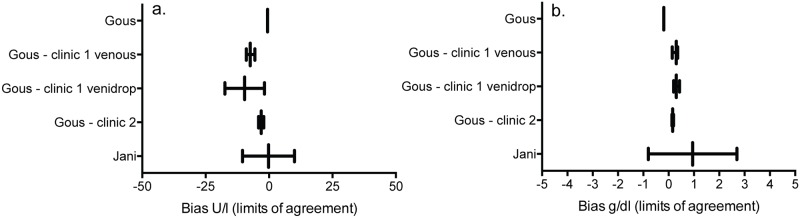
Forest plots of point-of-care ALT and hemoglobin diagnostic accuracy performance by non-laboratory staff. (a) point-of-care ALT testing by non-laboratory staff compared to conventional laboratory-based testing by professional laboratory staff; (b) point-of-care hemoglobin testing by non-laboratory staff compared to conventional laboratory-based testing by professional laboratory staff.

Three studies compared the performance of alanine aminotransferase (ALT) and hemoglobin enumeration tests operated by non-laboratory staff with conventional laboratory-based technologies operated by laboratory professionals [26–28]. Non-laboratory staff operated both tests comparably to conventional laboratory-based technologies operated by laboratory professionals (Fig 3a and 3b). A semi-quantitative, visual point-of-care ALT assay performed by nurses had a sensitivity and specificity of 87% and 77%, respectively, compared to a conventional laboratory-based technology operated by laboratory professionals [46]. Finally, one study reviewed the performance of creatinine and lactate testing by non-laboratory staff at two separate clinics [27]. Creatinine testing had mean bias values of -4.5 umol/L (95% CI: -2.09 –-6.42) and -5.5 umol/L (95% CI: -4.49 –-6.42), while lactate testing had mean bias values of 0.01 mmol/L (95% CI: -0.1–0.13) and 1.1 mmol/L (95% CI: 1.04–1.18).

### Quality of testing

**Fig 4:**
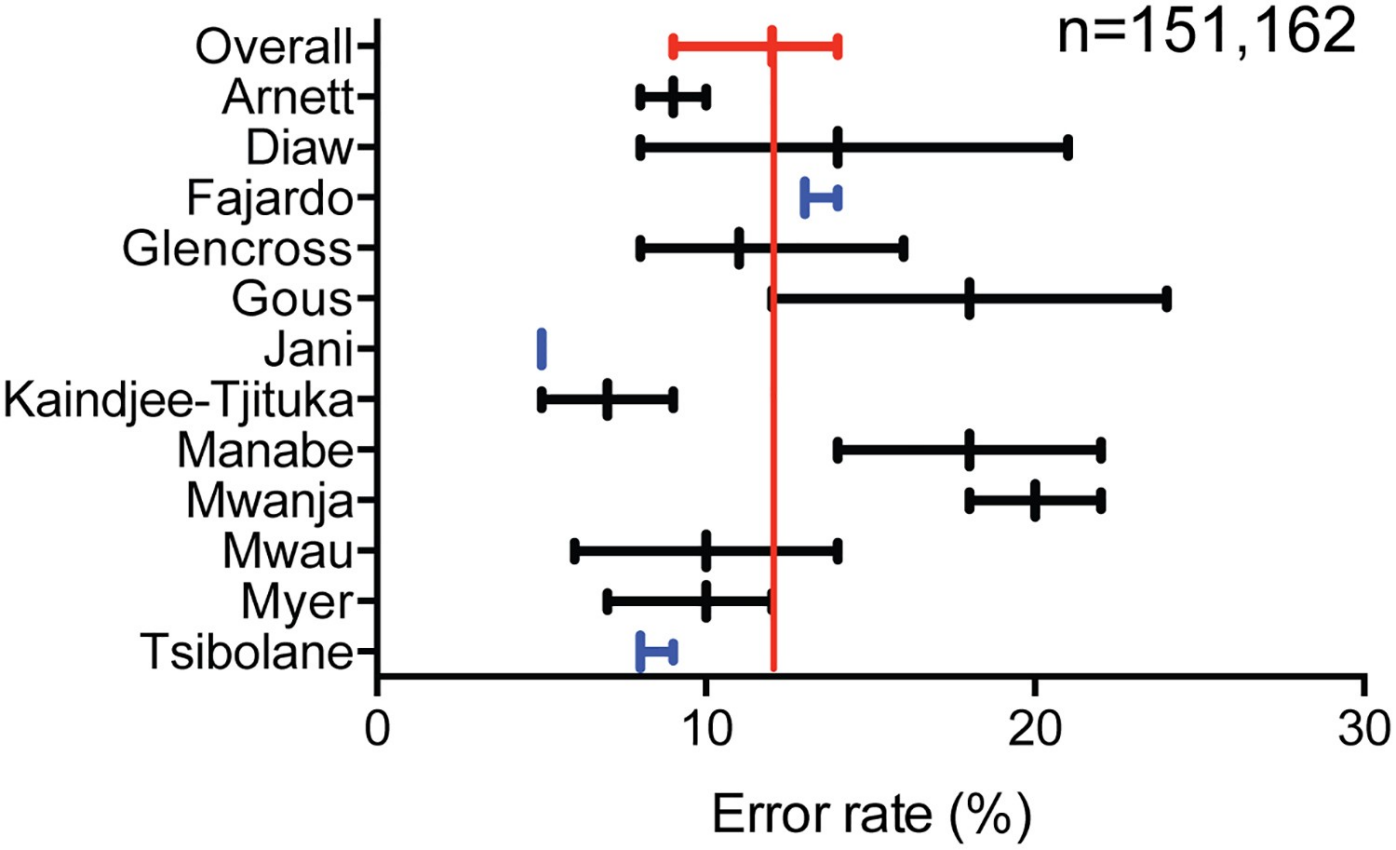
Error rates for point-of-care CD4 technologies operated by non-laboratory staff. Red forest plot and line indicate the overall pooled error rate. Blue forest plots indicate studies focused on program-wide, routine testing.

We further sought to understand the proportion of test errors (device and operator) encountered when technologies were used and specimens collected by non-laboratory staff. Only error rates from point-of-care CD4 technologies were reported in the included studies [15,20,21,25,30,36,40–42]. The proportion of error rates across studies for point-of-care CD4 was 12% (95% CI: 9–14%) (Fig 4). Unfortunately, error rates for conventional laboratory-based technologies operated by laboratory professionals were not included in any study, preventing comparison; however, the proportion of point-of-care CD4 errors was below 10% for two of the three studies where programmatic, routine point-of-care CD4 testing was performed [22,29,52]. The remaining nine studies reported point-of-care CD4 error rates from diagnostic accuracy evaluations and had significantly smaller sample sizes. Furthermore, additional studies found error rates using the Cepheid Xpert MTB/RIF were 17% (95% CI: 11–25%) and Alere q Detect early infant diagnosis were 9.24% when operated by non-laboratory staff [14,51].

### Acceptability of testing by non-laboratory staff

Eight studies assessed the acceptability and ease-of-use for testing operated by non-laboratory staff [15,16,23,24,30,35,38,49]. The measures of acceptability were heterogenous making summary statistics challenging. One study observed an ease-of-use score for task-shifting point-of-care CD4 testing between 1.7–3 using a scale of 1–5 (5 being very difficult) and health care worker trust in the test was measured at between 82–100% [15]. Another study found an odds ratio of 1.9 (1.1–3.3) for more rational use of higher-level clinical staff time with the introduction of point-of-care CD4 testing operated by lower-level staff instead [24]. Furthermore, 94.7% (95% CI: 92.9–95.9%) of lay health workers rated the point-of-care CD4 technology favorably [30]. Point-of-care viral load testing was found to be easy, or very easy, to use by all non-laboratory staff, while 85% of questionnaire respondents indicated that point-of-care viral load testing was suitable or very suitable for non-laboratory staff [38]. Ninety percent of non-laboratory staff identified that a syphilis rapid diagnostic test was easy to use [16], while antenatal care staff scored the dual HIV/syphilis rapid diagnostic test 2.41 (out of 3) for ease of use and 2.27 (out of 3) for ease of interpretation [44].

### Acceptability specimen collection by non-laboratory staff

Additionally, 58% of non-laboratory staff indicated that preparing dried blood spot specimens for viral load was very easy, while 43% indicated that the specimen collection was easy [23]. Eighty-five percent of respondents indicated that dried blood spot preparation was suitable for non-laboratory staff. A 98% success rate of finger-prick blood specimen collection by nurses was observed in South Africa [35]. Finally, one study found that sputum collection in ANC wards for tuberculosis testing was acceptable, but there were some concerns over staff availability, waiting times, and overload [49].

## Discussion

This review provides evidence of the ease-of-use, acceptability, and accuracy of task shifting to support access to specific laboratory tests in HIV programs. The introduction of point-of-care technologies as well as easy to use and/or stable specimen collection technologies, such as dried blood spot filter paper, accordingly allow for task shifting and decentralization of these clinical tasks. This review found that non-laboratory staff operated point-of-care testing comparably to laboratory professionals operating the same point-of-care test in the laboratory (mean bias +/- <15 cells/ul). Some variability, however, was observed comparing the performance of point-of-care CD4 testing by non-laboratory staff to conventional laboratory-based technologies by laboratory professionals (mean bias +/- < 55 cells/ul). These results are consistent with the fact that test variability can be expected even within the same technology and health care cadre; however, indicative that weaker performance may have been caused by the technology rather than the health care worker cadre performing the testing. Comparable performance was also seen with syphilis, cryptococcal diagnosis as well as ALT and hemoglobin testing, though the number of studies and sample sizes were small for each.

WHO recently recommended task shifting of HIV testing services to lay counselors [9], following a systematic review which found that uptake of testing doubled with task shifting of testing to lay counselors as well as comparable performance between lay counselors and laboratory staff in terms of accurate diagnoses [6,7]. Furthermore, this review found high patient satisfaction of HIV testing experiences when tested by lay counselors. Similarly, studies and guidelines have been published confirming the utility, impact, and non-inferiority of task shifting to deliver antiretroviral therapy [1,5,8], while this review provides and adds a diagnostic viewpoint. This systematic review highlights that task shifting for other laboratory tests is likely to be comparably valuable for increased patient access and decentralization. Additionally, health care facility staff and patient familiarity with task shifting of HIV rapid testing and same day test result delivery should allow for faster uptake of wider diagnostic task shifting.

Several studies reviewed the acceptability and ease-of-use of point-of-care technologies and specimen collection from the perspective of non-laboratory staff. All found that point-of-care testing and specimen collection were easy to perform and acceptable for non-laboratory staff. Furthermore, the acceptability of task shifting from the perspective of health workers is evidenced by widespread implementation of point-of-care testing and specimen collection performed by non-laboratory staff [59,60]. Dried blood spot sample collection for early infant diagnosis, for example, has been significantly decentralized and task-shifted to non-laboratory staff across sub-Saharan Africa and Southeast Asia [59,61,62]. A recent systematic review reported that within 12 studies, 90% of patients accepted point-of-care CD4 testing in primary health care and community settings [63].

Diagnostic assays have differing complexity and may vary in their suitability and potential placement based on health care facility infrastructure, human resource capacity, result interpretation, device and third-party equipment requirements, and reagent stability [13]. For example, rapid diagnostic tests or lateral flow assays generally do not require cold storage of reagents, electricity, specialized laboratory skills such as precise measurements using a pipet, daily calibration of devices, or centrifugation of specimens. Alternatively, there are device-based technologies that require some electricity, while others may require consistent electricity, temperature controlled rooms, precise measurements of specimens or reagents, or even plasma separation of whole blood using third-party procured centrifuges. Such technology characteristics and requirements may limit decentralization, the extent of task shifting, and thus patient access to testing.

While this review focused on HIV-related tests and specimen collection, the same principles, impact, and benefits, will likely apply beyond HIV programs. Task shifting of syphilis, hemoglobin, malaria, and/or hepatitis testing in antenatal facilities, for example, using rapid or point-of-care technologies by midwives could significantly expand access to these critical diagnostics. Additional tests, specimens, and program areas should be considered for task shifting as is feasible and beneficial, primarily to the patients and clinical delivery of optimal care.

As with task shifting of HIV treatment, the success of laboratory task shifting for supporting HIV care will rely on careful and transparent test and product selection processes, training, and quality monitoring and mentorship. Understanding patient volumes, testing needs, and health care facilities’ characteristics, such as available infrastructure and human resource capacity, will allow for appropriate selection of testing technologies that best fit each health care facility. Significant decentralization of any service, including task shifting of testing, will require careful training of health care facility staff prior to implementation. Furthermore, this approach should be supported by national policy, and this should stipulate the need for adequate training and supervision. Continuous monitoring and mentorship will ensure that challenges and corrective actions are quickly and appropriately managed. Ensuring these processes are implemented with task shifting into the national testing policies will support improved access to quality diagnostic testing and clinical patient management.

This review has several limitations. Most studies compared the point-of-care technology operated by non-laboratory staff with a conventional laboratory-based technology operated by laboratory professionals. The data generated thus far suggest task shifting specimen collection and point-of-care testing to non-laboratory staff provides comparable performance to laboratory professionals; however, additional studies with direct comparisons would be beneficial to further support the conclusions. Additionally, few studies looked at the diagnostic accuracy of task-shifting with other (non-CD4) test types and while some trends can be drawn, the conclusions would benefit from additional work, including other and new assays such as hepatitis C, hepatitis B core antigen, and human papillovirus. Further, as our review looked at all non-HIV rapid testing task-shifting, most studies focused on device-based assays. Comparisons between non-HIV testing laternal flow assays and device-based assays could not be made. Furthermore, search terms and engines may not have been exhaustive and could have missed studies eligible for inclusion. While retrospective studies provided compelling evidence, only one randomized control trials was included in the systematic review. Included studies had variable study objectives, different test types utilized, and various health care cadres, making comparisons and a meta-analysis difficult. Furthermore, most diagnostic accuracy studies had small sample sizes and, therefore, correspondingly wide confidence intervals that prevented strong conclusions. Finally, whilst it is critical to understand that diagnostic accuracy does not suffer with task shifting to non-laboratory staff, a primary objective of task shifting is to provide great access to faster test results, better overall quality care, and a more efficient system. However, no studies provided data on the timing and retention of patients along the cascade of care or cost-effectiveness of task-shifting specimen collection and/or testing.

Additional studies to more carefully determine the cause of diagnostic accuracy variability would be useful. For example, the level of variability expected within and between technologies. Furthermore, while studies have observed significant patient impact when implementing point-of-care CD4 technologies [64], it would be useful to better understand and weigh the benefits and harms of decentralization and task shifting with possible loss in testing quality, if observed.

An expert panel considered the findings of this review during the revision of the *2016 WHO guidelines on the use of antiretroviral drugs for treating and preventing HIV infection*. These updated guidelines recommend, as good practice, that trained supervised non-laboratory staff, including laypersons, can undertake blood finger prick for sample collection [12]. Incorporating task shifting for diagnostic testing and specimen collection into national policy will allow for greater decentralization and increased access of testing services as well as further support decentralized antiretroviral therapy management.

## Supporting information

S1 Fig Search terms for task shifting for sample collection and diagnostic test. (DOCX)

S2 Fig QUADAS-2 quality assessment summary of included studies. (TIF)
