# Supplementary material for: Performance of non-laboratory staff for diagnostic testing and specimen collection in HIV programs: A systematic review and meta-analysis
Source: PLoS One. 2019 May 2;14(5):e0216277. doi: 10.1371/journal.pone.0216277 (PMC6497381; doi:10.1371/journal.pone.0216277)
Supplement: S1 Fig — (DOCX) [file pone.0216277.s001.docx]

Search terms for task shifting for sample collection and diagnostic test (F4.2)

**EMBASE**

(HIV OR “HIV infections” OR hiv OR hiv-1 OR hiv-2 OR hiv1 OR hiv2 OR “hiv infect” OR “hiv infected” OR “hiv infection” OR “human immunodeficiency virus” OR “human immunedeficiency virus” OR “human immuno-deficiency virus” OR “human immune-deficiency virus” OR “acquired immunodeficiency syndrome” OR “acquired immunedeficiency syndrome” OR “acquired immuno-deficiency syndrome” OR “acquired immune-deficiency syndrome”)

AND

(non-laboratory OR professional OR “lay worker” OR provider OR counselor OR nurse OR health care worker OR “health care staff” OR vaccinator OR triage OR “clinical officer” OR task-shift OR task-shifting OR “task shift” OR “task shifting” OR “community health worker” OR “skill mix” OR “integration of tasks” OR “service delivery” OR “health services accessibility” OR volunteer OR voluntary OR “community care giver” OR “primary health care team” OR cadre OR midwife OR nurse assistant OR clinician OR physician OR non-physician OR “task sharing” OR “task share” OR “health auxiliary” OR midwife)

AND

(specimen OR test OR sample OR collect OR extract OR perform OR process)

AND

(blood OR venipuncture OR capillary OR heel-stick OR “heel stick” OR heel-prick OR “heel prick” OR finger-stick OR “finger stick” OR finger-prick OR “finger prick” OR DBS OR “dried blood spot” OR hemoglobin OR Hb OR “point of care” OR point-of-care OR POC OR rapid OR Alere OR Pima OR FACSPresto OR Daktari OR mBio OR Omega OR Burnet OR Partec OR PointCare OR Guava OR Millipore OR Cepheid OR GeneXpert OR Northwestern OR NW OR Lynx OR EID OR “early infant diagnosis” OR “DNA PCR” OR “RNA PCR” OR “viral load” OR VL OR CD4 OR CD4+ OR “T cell” OR “helper cell” OR “cluster of differentiation 4” OR RTK OR diagnosis OR diagnostic OR diagnostics OR diagnose OR screen OR screening OR screened OR laboratory OR creatinine OR Roche OR Liat OR SAMBA)

AND

(accuracy OR performance OR “technical evaluation” OR LTFU OR retention OR linkage OR attrition OR TAT OR “turnaround time” OR “time from test to result” OR “result returned” OR “results returned” OR “time to care” OR “time to refer” OR “time to referral” OR “time to referrals” OR “time to ART” OR “time to ARTs” OR “time to care” OR “time to drug” OR “time to drugs” OR “TAT to ART” OR “TAT to ARTs” OR “adverse event” OR “adverse events” OR morbidity OR mortality OR illness OR disease OR death OR acceptable OR acceptability OR care OR follow-up OR “follow up” OR “result notification” OR cost OR cost-effectiveness OR DALY OR ICER OR affordability OR affordable OR feasibility OR feasible OR acceptability OR acceptable OR confidential OR confidentiality OR safety)

AND

Publication date after January 1, 2005

**MEDLINE**

(HIV [MeSH] OR HIV infections [MeSH] OR hiv [tw] OR hiv-1* [tw] OR hiv-2* [tw] OR hiv1 [tw] OR hiv2 [tw] OR hiv infect* [tw] OR human immunodeficiency virus [tw] OR human immunedeficiency virus [tw] OR human immuno-deficiency virus [tw] OR human immune-deficiency virus [tw] OR ((human immun*) AND (deficiency virus [tw])) OR acquired immunodeficiency syndrome [tw] OR acquired immunedeficiency syndrome [tw] OR acquired immuno-deficiency syndrome [tw] OR acquired immune-deficiency syndrome [tw] OR ((acquired immun*) AND (deficiency syndrome [tw])) OR "sexually transmitted diseases, viral" [MESH:NoExp])

AND

(non-laboratory OR professional OR lay worker OR provider OR counselor OR nurse OR health care worker OR health care staff OR vaccinator OR triage OR clinical officer OR task-shift* OR task shift* OR community health worker OR skill mix OR integration of tasks OR service delivery OR health services accessibility OR volunteer OR voluntary OR community care giver OR primary health care team OR cadre OR midwife OR nurse assistant OR clinician OR physician OR non-physician OR task shar* OR health auxiliary OR midwife)

AND

(specimen OR test OR sample OR collect OR extract OR perform OR process)

AND

(blood OR venipuncture OR capillary OR heel-stick OR heel stick OR heel-prick OR heel prick OR finger-stick OR finger stick OR finger-prick OR finger prick OR DBS OR dried blood spot OR hemoglobin OR Hb OR point of care OR point-of-care OR POC OR rapid OR Alere OR Pima OR FACSPresto OR Daktari OR mBio OR Omega OR Burnet OR Partec OR PointCare OR Guava OR Millipore OR Cepheid OR GeneXpert OR Northwestern OR NW OR Lynx OR EID OR early infant diagnosis OR DNA PCR OR RNA PCR OR viral load OR VL OR CD4 OR CD4+ OR T cell OR helper cell OR cluster of differentiation 4 OR RTK OR diagnosis OR diagnostic* OR diagnose OR screen* OR laboratory OR creatinine OR Roche OR Liat OR SAMBA)

AND

(accuracy OR performance OR technical evaluation OR LTFU OR retention OR linkage OR attrition OR TAT OR turnaround time OR time from test to result OR result returned OR results returned OR time to care OR time to refer* OR time to ART* OR time to care OR time to drug* OR TAT to ART* OR adverse event* OR morbidity OR mortality OR illness OR disease OR death OR accept* OR care OR follow-up OR follow up OR result notif* OR cost OR cost-effectiveness OR DALY OR ICER OR affordab* OR feasib* OR accept* OR confident* OR safety)

AND

Publication date after January 1, 2005

**PubMed**

(HIV OR HIV infections OR hiv OR hiv-1* OR hiv-2* OR hiv1 OR hiv2 OR hiv infect* OR human immunodeficiency virus OR human immunedeficiency virus OR human immuno-deficiency virus OR human immune-deficiency virus OR ((human immun*) AND (deficiency virus)) OR acquired immunodeficiency syndrome OR acquired immunedeficiency syndrome OR acquired immuno-deficiency syndrome OR acquired immune-deficiency syndrome OR ((acquired immun*) AND (deficiency syndrome)) OR sexually transmitted diseases, viral)

AND

(non-laboratory OR professional OR lay worker OR provider OR counselor OR nurse OR health care worker OR health care staff OR vaccinator OR triage OR clinical officer OR task-shift* OR task shift* OR community health worker OR skill mix OR integration of tasks OR service delivery OR health services accessibility OR volunteer OR voluntary OR community care giver OR primary health care team OR cadre OR midwife OR nurse assistant OR clinician OR physician OR non-physician OR task shar* OR health auxiliary OR midwife)

AND

(specimen OR test OR sample OR collect OR extract OR perform OR process)

AND

(blood OR venipuncture OR capillary OR heel-stick OR heel stick OR heel-prick OR heel prick OR finger-stick OR finger stick OR finger-prick OR finger prick OR DBS OR dried blood spot OR hemoglobin OR Hb OR point of care OR point-of-care OR POC OR rapid OR Alere OR Pima OR FACSPresto OR Daktari OR mBio OR Omega OR Burnet OR Partec OR PointCare OR Guava OR Millipore OR Cepheid OR GeneXpert OR Northwestern OR NW OR Lynx OR EID OR early infant diagnosis OR DNA PCR OR RNA PCR OR viral load OR VL OR CD4 OR CD4+ OR T cell OR helper cell OR cluster of differentiation 4 OR RTK OR diagnosis OR diagnostic* OR diagnose OR screen* OR laboratory OR creatinine OR Roche OR Liat OR SAMBA)

AND

(accuracy OR performance OR technical evaluation OR LTFU OR retention OR linkage OR attrition OR TAT OR turnaround time OR time from test to result OR result returned OR results returned OR time to care OR time to refer* OR time to ART* OR time to care OR time to drug* OR TAT to ART* OR adverse event* OR morbidity OR mortality OR illness OR disease OR death OR accept* OR care OR follow-up OR follow up OR result notif* OR cost OR cost-effectiveness OR DALY OR ICER OR affordab* OR feasib* OR accept* OR confident* OR safety)

AND

Publication date after January 1, 2005
