# Supplementary figures and images for: Performance of non-laboratory staff for diagnostic testing and specimen collection in HIV programs: A systematic review and meta-analysis
Source: PLoS One. 2019 May 2;14(5):e0216277. doi: 10.1371/journal.pone.0216277 (PMC6497381; doi:10.1371/journal.pone.0216277)

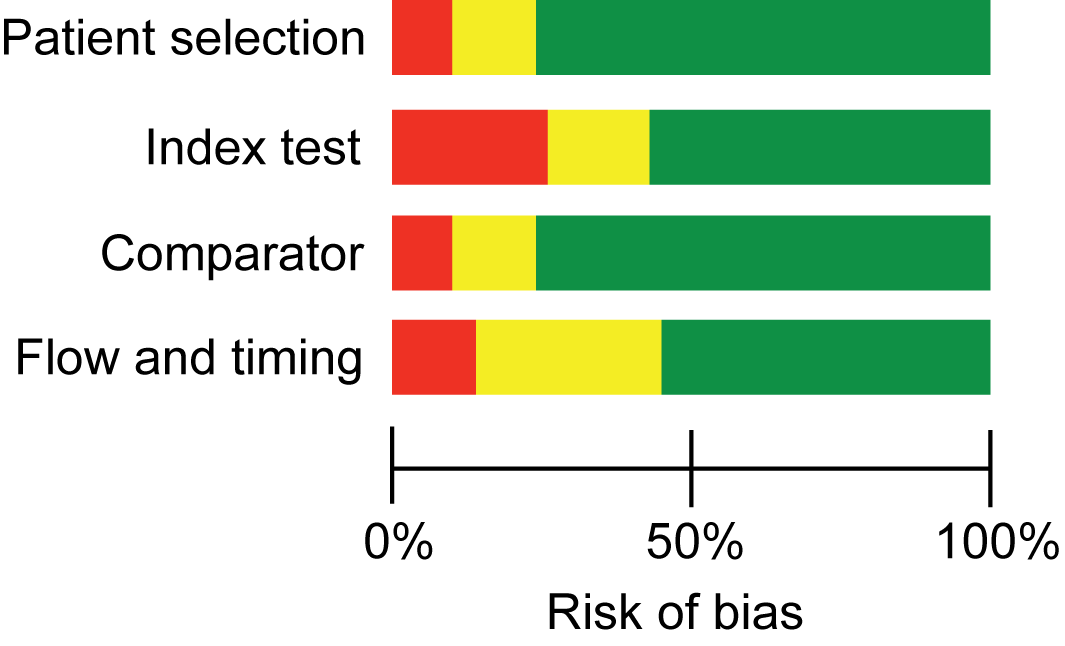

Supplement: S2 Fig — (TIF) [file pone.0216277.s002.tif]
